# Using CFD simulations to investigate the shear stress in hydrodynamic cavitation reactors coupled with experimental validation using colony count measurements

**DOI:** 10.1038/s41598-022-20349-7

**Published:** 2022-10-27

**Authors:** Máté Polgár, Charu Agarwal, Parag Gogate, Gábor Németh, Levente Csóka

**Affiliations:** 1 Faculty of Wood Engineering and Creative Industry, University of Sopron, 9400 Sopron, Hungary; 2 Aqua-Filt Ltd., 9400 Sopron, Hungary; 3 Department of Chemical Engineering, Indian Institute of Technology, Hauz Khas, New Delhi, 110016 India; 4 Department of Chemical Engineering, Institute of Chemical Technology, Matunga, Mumbai, 400019 India; 5 ELTE Eötvös Loránd University, Faculty of Informatics, 1053 Budapest, Hungary

**Keywords:** Isolation, separation and purification, Chemical engineering

## Abstract

The current work investigates the shear stress distribution in hydrodynamic cavitation reactors with two different geometries using CFD simulations. Venturi type (positive geometry) and bore (negative geometry) were used to induce cavitation. Experimental validation of the predictions from simulations was also conducted by calculating the reduction rate in the colony count of *Legionella pneumophila*, a pathogenic bacterial strain. Both the numerical and experimental studies revealed the significant influence of the shape of the cavitation-inducing geometry on the flow characteristics and the distribution of shear stress. The simulation data indicated high shear stress formation in the positive geometry as a venturi, with the cavitation ranges for the two reactors being far apart from each other. The experimental study also confirmed that the flow conditions in the venturi-type reactor were more favourable compared to the bore geometry, resulting in a bacterial reduction efficiency as high as 99.98%. It was clearly demonstrated that the geometry of the cavitating device plays a crucial role in deciding the shear stress and its efficacy for the desired applications as per the predictions of the simulation model validated by the experimental results.

## Introduction

Cavitation refers to the formation, growth, and subsequent collapse of bubbles in a liquid medium and can occur in two ways, by alterations in sound pressure field (acoustic cavitation) or by pressure change based on changes in geometry (hydrodynamic cavitation)^1^. The phenomenon of hydrodynamic cavitation has been easily explained by Bernoulli’s principle^2^, which states that as the velocity of a fluid increases, its static pressure decreases. If the degree of pressure drop is enough to reach the saturation vapor pressure at a given temperature, the liquid is converted into the vapor phase, resulting in the formation of gas/vapor-filled cavities. Subsequently, as the area available for flow increases, the pressure recovers and the cavities are subjected to oscillating pressure fields, leading to violent collapse of these cavities. In a cavitation reactor, a significant number of cavities are created every millisecond^3^. Transient cavitational collapse induces high-pressure shock waves and free radicals with temperatures of up to 10,000 K, causing physical and chemical changes in the target matrix^4,5^ and driving many process intensification applications. The phenomenon of cavitation can indeed be applied in several areas of process intensification, such as food processing, emulsification, extraction, biofuels, and environmental remediation, especially wastewater treatment^5^. The extreme conditions and the violent collapse of cavities favour processes such as the reduction of bacterial colony ^6^ and the destruction of wastewater contaminants^7–9^. The use of hydrodynamic cavitation can indeed lead to significant process intensification benefits, such as a reduction in time, higher rates of processing, and the use of less forcing conditions in terms of temperature or pressure^10,11^.

A theoretical understanding of the cavitation phenomena in terms of the pressure field distribution is important and required for the effective design of cavitational reactors that can be applied at a larger scale of operation for process intensification. For example, Sutkar et al. described the three-dimensional wave equation and numerically studied the acoustic effects in a liquid medium^12^ based on the wave equation that explains the propagation of sound waves through the liquid used to generate acoustic cavitation. Ultrasound results in periodic pressure changes in the fluids, which is the acoustic pressure. The advantage of the acoustic pressure is that it can be used in the bubble dynamics equation, a second-order nonlinear differential equation describing the dynamics of a bubble, for predicting the intensity of cavitation.

Hydrodynamic cavitation is generated by pressure variation obtained by passing a liquid through a constriction, resulting in an increase in liquid velocity at the expense of local pressure^4,5,13^. Process parameters such as energy input, time, temperature, fluid quality, and the geometry of the flow equipment can largely influence the effectiveness of hydrodynamic cavitation technology^14–17^. Hydrodynamic cavitation requires inexpensive devices to operate, is energy-efficient, and has great potential for industrial-scale process intensifications. The threshold of cavitation formation can be characterized by the cavitation number. This dimensionless factor is the ratio of the dynamic and the static components^18^. Significant cavitational effects occur at low cavitation numbers (< 1) as also reported in literature^19,20^. However, higher cavitation numbers (between 2 and 4) may also result in cavitation, probably due to the presence of dissolved gases or impurities in the liquid medium^21^.

Noticeable works have recently been published focusing on the influence of cavitation-inducing geometry and operating conditions on the flow characteristics in hydrodynamic cavitation reactors^22,23^. Many studies have carried out both experimental and simulation-based research to study hydrodynamic cavitation^24–28^. The most commonly used geometric designs in hydrodynamic cavitation, such as the venturi and orifice plates, have also been applied in theoretical computations for the prediction of cavitational characteristics. For instance, the influence of upstream and downstream pressures, as well as geometric parameters (throat diameter, throat length, and diffuser angle), on the flow performance of cavitating venturi has been studied^24^. Another work found a significant effect of the geometric design of a venturi on cavitation inception and microbubble generation^22^. Similarly, different orifice designs have been analysed in the modelling of hydrodynamic cavitation^23^. Rotational hydrodynamic homogenizers have also been used to create cavitation conditions and for subsequent applications. For example, a high-speed homogenizer has been used to disinfect water at different speeds^18^ to demonstrate effective performance as a function of operating speed.

Most of the works on hydrodynamic cavitation reactors have largely investigated the effect of critical geometric parameters limited to the specific geometry (orifice or venturi or the rotational equipment) on cavitation performance using the existing simulation models^16,22,23,25,26,29^. There have been some recent studies dealing with basic understanding into the inception of cavitation or quantifying the extent of cavitation as a function of operating conditions, mainly the pressure drop across the cavitating device^30^. Another new approach for modelling has been to use artificial neural networks as reported by Ranade et al*.* for the case of pretreatment of biomass with an objective of enhancing the biogas yield and for wastewater treatment^31^. Analysis of literature revealed that, little attention has been paid to the effect of the cavitation-inducing geometry itself on the shear stress distribution in cavitation reactors. Considering these lacunae, the present study aims to investigate how the difference in cavitational geometry affects the cavitation efficiency and the shear stress in the two types of cavitation reactors applied in the work of Badve et al*.*^32^ and Šarc et al*.*^33^ using numerical simulation. Experiments were also carried out to validate the simulation results by comparing the reduction rate in the colony count of *Legionella pneumophila*. The specific application of microbial disinfection has been selected considering the fact that controlling mechanism for disinfection is shear stress and hence the theoretical simulations provide a useful correspondence with the actual investigated applications. In addition, the knowledge of the shear distribution in different types of reactors can provide reference data for hydrodynamic cavitation and can prove fundamental in enhancing the cavitation efficiency for large-scale industrial applications.

## Experimental and model details

### Structure and settings of hydrodynamic cavitation equipment

**Figure 1:**
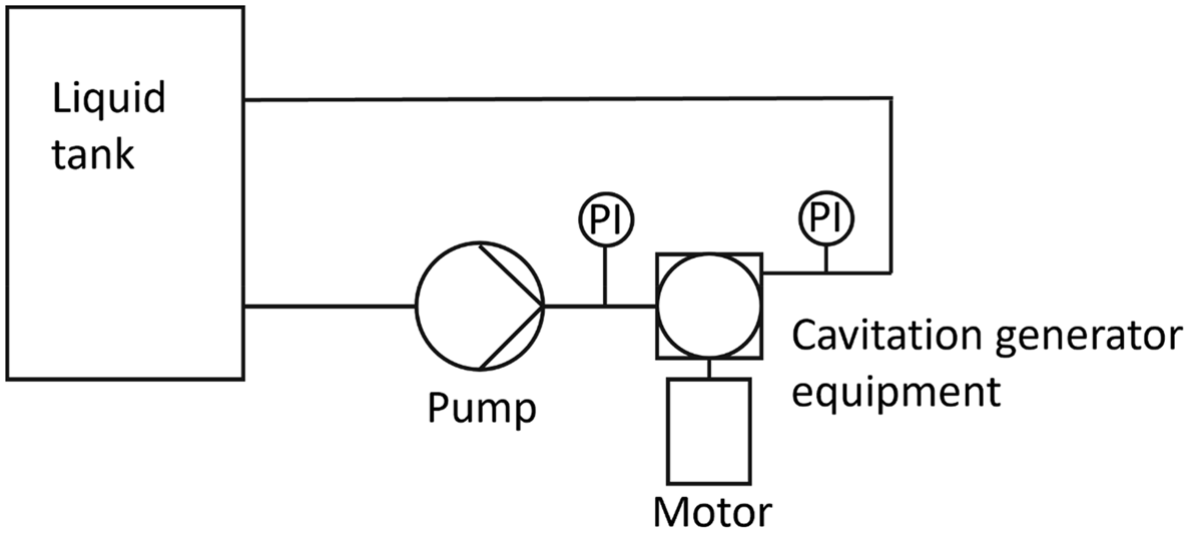
Schematic illustration of the water treatment system.

Figure 1 illustrates the treatment system, including tank contents, cavitation units and an electric motor to drive the cavitating device. The basic water treatment system scheme is similar in the two rotational cavitation experimental configurations (details below Fig. 1), but the geometric designs of the hydrodynamic cavitation reactors are different.

Badve et al*.* demonstrated the use of a rotational cavitation device (hereafter called BCR) for iodine liberation and applied numerical simulations for a better understanding of cavitation phenomena^32^. The study dealt with the shear stress and shear rate of Newtonian fluids at different rotation speeds, and its validation by the measured iodine liberation in the liquid. The authors found a correlation between the characteristic shear ratio and iodine liberation. In the cavitation reactor applied in this work, the motor speed could be changed as the electrical system included a frequency inverter, where the adjustable speeds ranged from 900 to 2800 rpm, and the motor power was 4 kW^32^.

Šarc et al*.* applied another configuration of a rotational cavitation reactor (hereinafter called SCR) to induce cavitation phenomena^33^ and experimentally investigated the effect of cavitation on various bacterial colonies in different structural designs. The authors compared the venturi and bore-design based rotational cavitation reactor systems and found that the venturi-designed rotational cavitation reactor had a higher reduction rate for *Legionella pneumophila*. In this cavitation reactor, the motor speed was constant at 9025 rpm, and the motor power was 280 W. Rotating discs were mounted on the motor shafts, which could be rotated to create cavitation fields. The authors investigated the effect of different flow parameters on bacterial colony count reduction using three bacterial strains, viz., *Legionella pneumophila*, *Escherichia coli*, and *Bacillus subtilis*^33^.

Since the present study addresses the measurement of *Legionella pneumophila* colony count only, the most favourable results on colony count reduction from the work of Šarc et al*.*^33^ were used in the simulation settings. The values of the physical and fluid parameters set during the experiment are summarized in Table 1.

**Table 1:** Experimental conditions of flow.

	Šarc’s setup	Badve’s experimental setup		
Speed of rotation (rpm)	9025	1500	2200	2800
Pressure (Pa)	200,000	150,000	150,000	150,000
Volumetric flow rate (m^3^/h)	0.12	2.8	2.8	2.8
Cavitation number	0.7	1.26	0.58	0.36

According to the results for *SCR*, the largest reduction is achieved in the supercavitation range. Table 1 shows the flow parameters set during each simulation, from which the cavitation numbers were calculated. *Legionella* colony count measurements were used to validate the simulations. Due to the high computational demands of the simulations, only the best degradation setting was modeled from the published *SCR* results, as presented in Table 1. In our study, three different motor speeds were defined, while the other flow parameters (pressure and volumetric flow) remained unchanged during the experiments. The liquid in both setups was water, so the fluid properties were the same: the density was 1000 kg/m^3^, the viscosity was 0.001 Pa s, and the temperature was 25 °C (vapor pressure of water at 25 °C is 3165 Pa). The sample volume was 500 L for *BCR* and 2 L for *SCR* setups.

### Geometric design of the rotor of SCR

**Figure 2:**
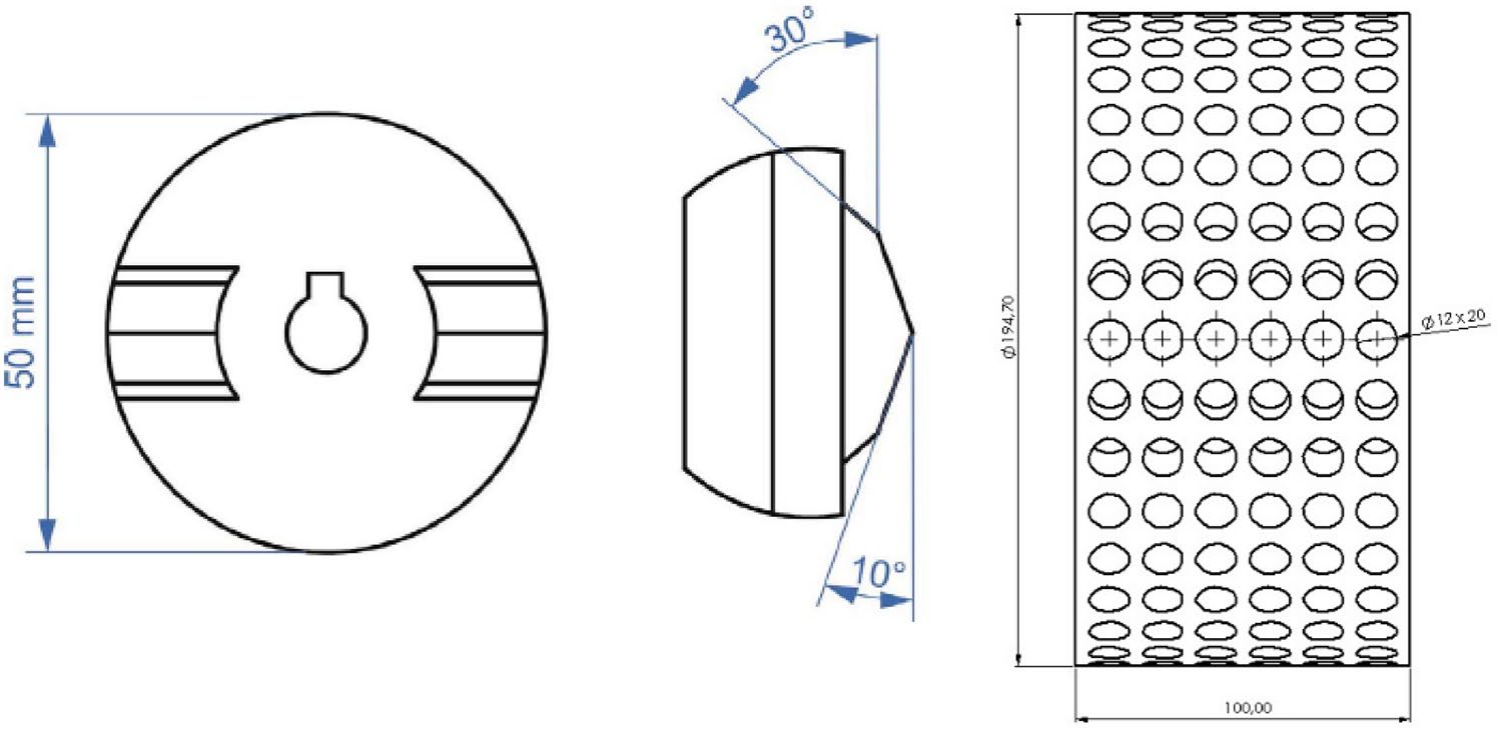
Rotors of *SCR* (left and middle)^33^ and *BCR* (right) equipment.

The cavitation inception and yield in venturi are greatly influenced by geometric parameters, including the throat length and diameter, as well as the convergent and divergent angles^27^. The design of the rotating disk in *SCR* is a “positive” geometry, as shown in Fig. 2 (left)^33^. Venturi geometry is machined on the face of a cylinder with two deflections. The diameter of the cylinder is 50 mm, while the deflection of the two teeth is 30° and 10°, respectively. Two “positive” geometries are machined on the face of the cylinder.

### Geometric details of the rotor of BCR

The fundamental difference between the two rotors is in the location and shape of the cavitation-creating geometry. Compared to *SCR*, in *BCR,* the cavitation-creating geometry is the bore, which can be considered a “negative” shape. The indentations, with a diameter of 12 mm and a depth of 20 mm, are located concentrically on the cylinder shell, as depicted in Fig. 2 (right). The distance between the indentations is 16 mm, and there are 34 indentations in a circular row. The mantle has seven circular rows of indentations, and so there are a total of 238 indentations on the cylinder.

### Theoretical model description

The Navier–Stokes equations are used to mathematically describe the flow of fluids and are governed by mass, momentum, and energy equations with no analytical solution. However, with numerical methods such as computational fluid dynamics (CFD), we can obtain approximate results about the nature of flow. In this study, the Solidworks Flow Simulation program was used as a simulation tool for calculations. The software solves the Navier–Stokes equation with consideration of the Favre-averaged Navier–Stokes equation for turbulent flows. The most commonly used k-ε model was applied in the work to simulate the mean flow characteristics in the turbulent conditions. The different applied model equations including the conservation of mass and momentum as well as the equations to predict the turbulent kinetic energy dissipation and its rate are given by Eqs. (1)–(4)^34^.

1 $$\frac{\partial \rho }{\partial t}+\frac{\partial }{\partial {x}_{i}}\left(\rho {u}_{i}\right)=0$$

2 $$\frac{\partial \rho {u}_{i}}{\partial t}+\frac{\partial }{\partial {x}_{i}}\left(\rho {u}_{i}{u}_{j}\right)+\frac{\partial p}{\partial {x}_{i}}=\frac{\partial }{\partial {x}_{i}}\left(\rho {\tau }_{ij}+{\tau }_{ij}^{R}\right)+{S}_{i}$$

3 $$\frac{\partial \rho H}{\partial t}+\frac{\partial }{\partial {x}_{i}}\left(\rho H{u}_{i}\right)=\frac{\partial p}{\partial {x}_{i}}+\frac{\partial }{\partial {x}_{i}}\left({u}_{j}\left(\rho {\tau }_{ij}+{\tau }_{ij}^{R}\right)+{q}_{i}\right)-{\tau }_{ij}^{R} \frac{\partial }{\partial {x}_{j}}\left({u}_{i}\right)+\rho \varepsilon +{S}_{i}{u}_{i}+{Q}_{H},$$

4 $$H=h+\frac{{u}^{2}}{2}+\frac{5}{3}k-\frac{{\Omega }^{2}{r}^{2}}{2}-{\Sigma}_{m}{h}_{m}{y}_{m}\;\;(\text{for} \; \text{rotational} \;\text{motion})$$

### Boundary conditions and simplified simulation models

The present study addresses two different types of hydrodynamic reactors applied by Šarc et al*.*^33^ and Badve et al*.*^32^. The CFD simulations were analyzed with two fluid connections, an inlet, and an outlet in both cases. These connections represent the boundary connections. The volumetric flow rate was defined at the input and pressure at the output, considering the experimental settings summarized in Table 1. In general, depending on the design, rules can be established to determine the actual cell number for the simulations.

Finite element method requires breaking of the computational domain (i.e. entire reactor area) into simple geometry such as triangle and tetrahedrons elements, which are described as mesh/grids. In both reactors, rotating subdomains were divided using tetrahedral elements. The computational domains involved were divided using the rotational invariant geometries and then the conservation equation solved on a rotating coordinate system to account for the indented cylinder. The house around is expressed in the fixed material coordinate system. An identity interface approach was applied to connect the rotating and standing zones together. The approach assumes that, at the interface, the flux continuity boundary condition is applied. To achieve a sufficient refined mesh, i.e., each bore must contain nearly equal numbers of cells, which was achieved by using a mesh generation tool. Since the direction of the propagation is generally not known beforehand, isotropic mesh calculation is useful. The number of elements in a sufficiently resolved mesh should be about two third of the degree of freedom (DOF). During the calculation, the necessary DOFs were 450,000–2,250,000 for a fully reliable solution.

Another practical requirement is that at least five cells in the “channel” must be defined in the flow direction (transverse direction). In the case of three-dimensional simulation, based on the values depending on the geometry to be examined, it is optimal at 300,000–1,500,000 cells^35^.

The basic simulation settings are based on the applied rotating field model. The *sliding mesh* model can be used because the *global rotating region* model is applicable for axisymmetric flow or in the averaging rotating region case of “smears”, for the streamlines along the outer mantle due to the perforated design.

**Figure 3:**
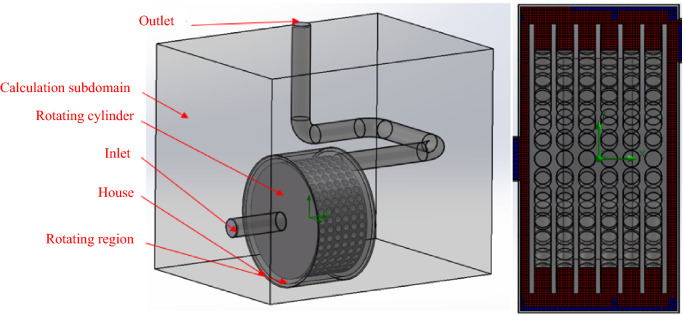
Simplified simulated representation and mesh settings of *BCR* equipment.

**Figure 4:**
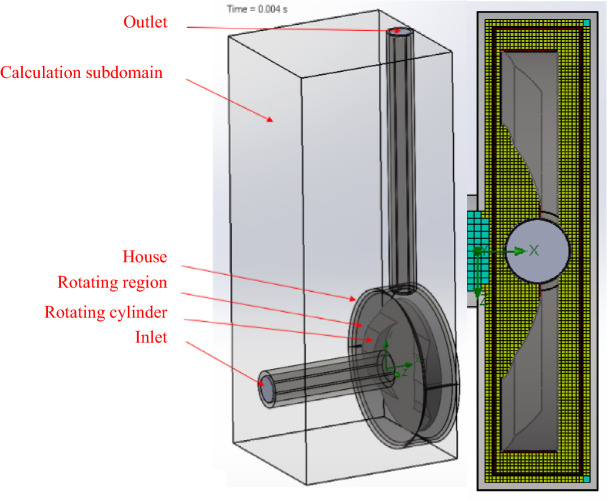
The simplified simulated representation and the mesh settings of the *SCR* equipment.

A simplified simulation model of *BCR* experimental equipment contains the main components for cavitation, which are the housing and the rotating cylinder (Fig. 3). In the design of the homogeneous mesh, due to the geometrically complicated design of *BCR* (Fig. 4 and Table 2), the total number of cells is 784,041 and the number of contacting fluid cells is 406,905. Table 2 describes the number of cells applied in the case of both BCR and SCR.

**Table 2:** Number of cells in the *BCR* and *SCR.*

	SCR	BCR
Total cells	427,815	784,041
Fluid cells	427,815	784,041
Fluid cells contacting solids	154,537	406,905

There are two “positive” shear elements on the rotating cylinder face in the case of SCR; the finite element mesh cell number based on the aforementioned meshing technique is less, with a total cell count of 427,815 and a solid surface contacting cell count of 154,357.

Due to the complexity of the models, the time step applied is 2 × 10^–5^ s. The defined target values are measured by the average global pressure, the average speed, the torque acting on the cylinder due to the flow (*x*, *y*, and *z* directions), and the shear stress (average and maximum) on the cylinder surface based on the instrumentation of the experimental equipment. According to Badve et al*.*, the shear stress on the cylinder surface is given by Eq. (5)^32^.

5 $$\tau =(\mu +{\mu }_{t})\frac{du}{dn}$$

where *µ* is the fluid dynamic viscosity (laminar), *µ*_*t*_ is the turbulent viscosity, *u* is the fluid velocity, *n* is the direction normal to the wall surface, and *du/dn* is defined at the wall surface.

To reach the convergence level, the stopping function of the simulation is governed by the convergence of the goal values, and a final closing condition is a maximum of 2500 iterations requiring around 14 h (1 iteration lasts around 20 s).

### Sampling and determination of *Legionella pneumophila*

The pathogenic bacterial strain *Legionella pneumophila* occurs in warm water systems and is known to cause a respiratory illness in humans called the “Legionnaires” disease. The experimental steps involving sampling, detection and interpretation of results were performed using the Legipid® *Legionella* Fast Detection kit^36^. The test principle combines magnetic immunocapture and enzyme-linked immunoassay with enzymatic colorimetric reaction. The protocol is based on the ISO 11731 standard for the detection and enumeration of *Legionella* in water.

#### Sample preparation

To prepare the test sample, the original water sample was filtered using a polycarbonate filter with a pore diameter of 0.40 μm. The filter was separated from the filtration system, cut into pieces and put into a flask containing eluent. The filter was eluted by shaking for 2 min, and the eluted sample was used for subsequent analysis.

#### Analysis

A homogeneous suspension of the capture reagent (immunomagnetic particles) was added to each cuvette. The test sample (previously filtered and eluted) was added to the test cuvette, taking care not to let fall any pieces of filter, while the eluent was added to the control cuvette. The cuvettes were closed with plugs. The cuvette holder was gently swayed every few minutes for 15 min to ensure uniform mixing. After 5 min, when the magnetic particles were retained, the supernatant was discarded by emptying the cuvettes on the opposite side to the magnets. Subsequently, the wash buffer was added to each cuvette, and it was shaken vigorously (without plugs) to resuspend the particles. After 3 min, the supernatant was discarded by emptying the cuvettes.

For marking, the enzyme-conjugated anti-*Legionella* antibody reagent was added to each cuvette followed by vigorous shaking. After 3 min, the supernatant was discarded by emptying the cuvettes. The wash buffer was added to each cuvette, and it was shaken vigorously (without plugs) to resuspend the particles. After 3 min, the supernatant was discarded, wash buffer was added, and the cuvettes were shaken.

For detection, the reagent with enzymatic cosubstrates was added to each cuvette and shaken gently for 2 min. This was followed by the addition of stop reagent to each cuvette and a 5 min pause to retain the magnetic particles.

#### Interpretation by optical reading

The control and test supernatants were transferred to their corresponding measurement units. The absorbance of a cuvette filled with distilled water was measured at 429 nm and set at zero. The absorbance of the supernatant of the control was measured at 429 nm as a reference and readjusted to zero. Finally, the absorbance of the supernatant of each test was measured at 429 nm.

If the relative absorbance readings of the samples were below the cut-off value, the result was negative and reported as “not detected”. If the relative absorbance readings of the samples were above the cut-off value, the result was positive and reported as “detected”. For positive results, the log_10_ of the relative absorbance was obtained. The concentration of *Legionella* in the volume was calculated in logarithm units first by entering the value of the log_10_ of the relative absorbance (A_r_) in the Eq. (6) as follows:

6 $${\text{y }} = { 2}.{3}0{\text{61 x }} + { 4}.{9815}$$

where x = log_10_ (A_r_) and y = log_10_ (CFU/volume examined).

The result for actual concentration in CFU/mL was obtained by applying the inverse transformation of the logarithm given by Eq. (7).

7 $${\text{Contamination }}\left( {{\text{CFU}}/{\text{volume examined}}} \right) \, = { 1}0^{{\text{y}}}$$

## Simulation and experimental results

### Simulation results

**Figure 5:**
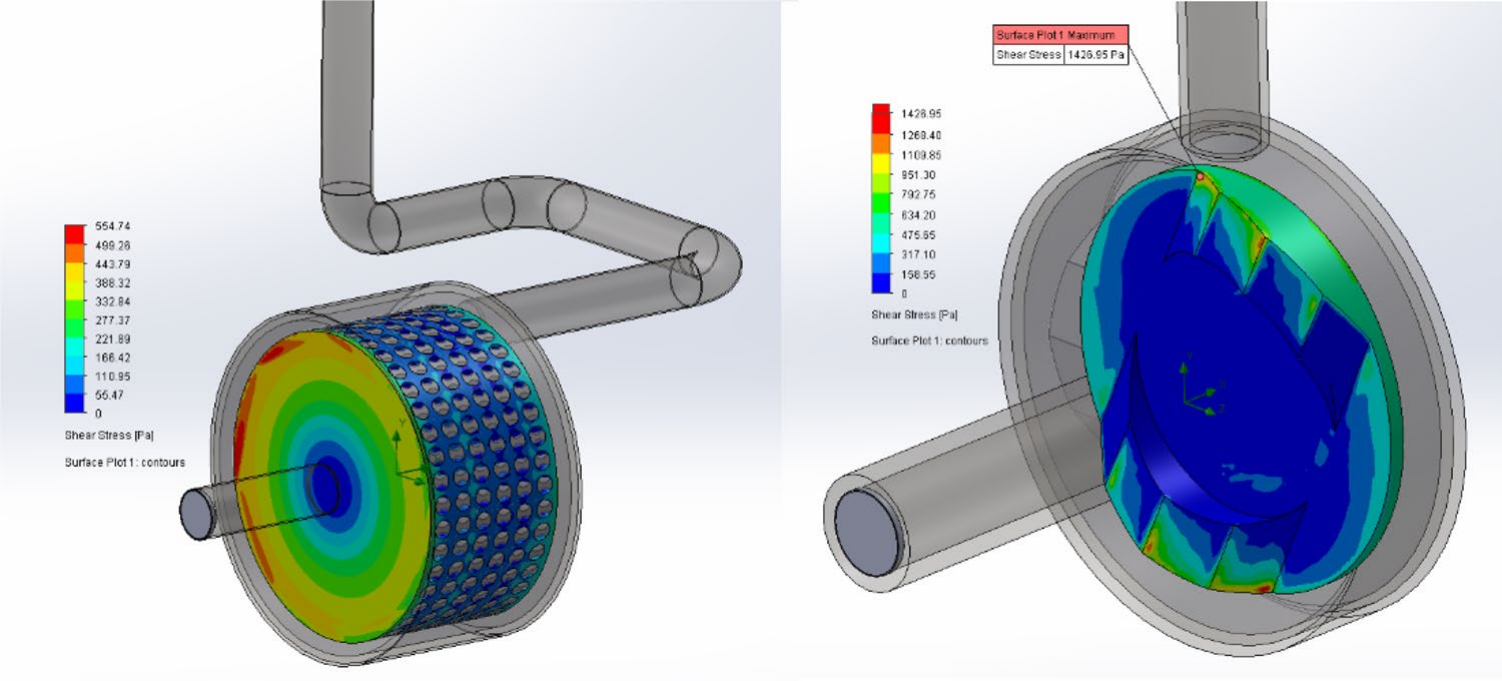
Shear stress distribution plot of SCR (left) and BCR (right).

Based on the study of Badve et al*.*^32^, computational resources were used to calculate the distribution of the shear stress on the surface. Figure 5 provides insight into the shear stress distribution on rotating geometries. The highest shear stresses developed at the inlet edges. High shear stress values were obtained at the “positive” edges, whereas low shear was created in the perforated design.

**Figure 6:**
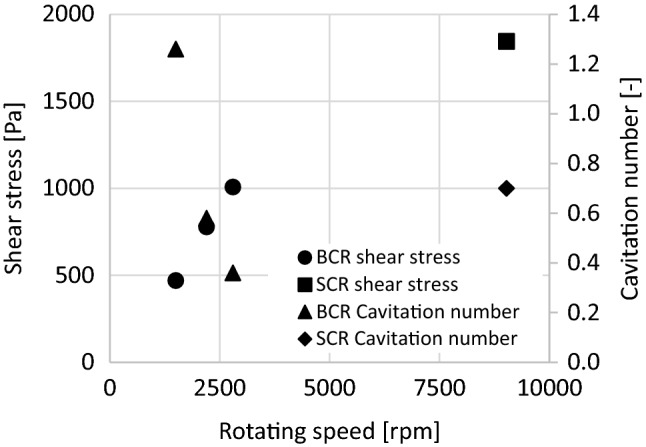
Cavitation number and shear stress values at different speeds.

Figure 6 shows the calculated maximum stress and cavitation number values at different speeds. The maximum shear stress was observed in the *BCR* design at 2200 rpm and in *the SCR design* at 9025 rpm.

During the simulations, the highest shear stress (1843.97 Pa) was obtained for the *SCR*. The *SCR* shear stress was 2 times higher than the *BCR*; however, the 20 times higher flow rate in the *BCR* resulted in nearly identical viable bacterial reduction explaining the superiority of SCR in terms of higher shear stress.

### Experimental results

**Figure 7:**
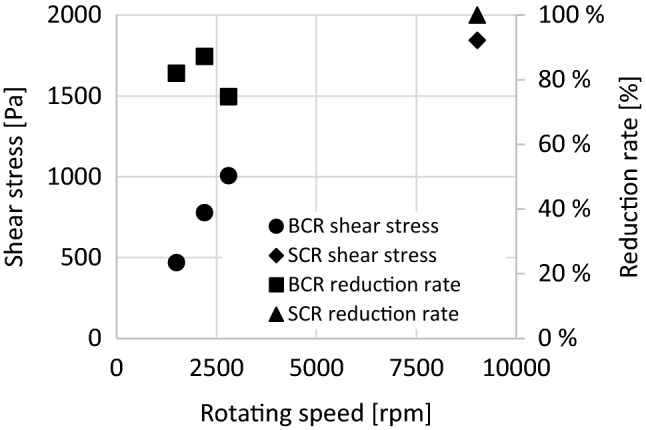
Shear stress and reduction rate values at different speeds.

Table 3 provides insight into the simulated shear stress values, the measured colony count values during the experiments, and the reduction rate between the control and treated samples (Fig. 7).

**Table 3:** Summary of results.

	SCR	BCR		
Rotating speed (rpm)	9025	1500	2200	2800
Pressure (Pa)	150,000	150,000	150,000	150,000
Volumetric flow rate (m^3^/h)	0.012	2.8	2.8	2.8
Cavitation number	0.7	1.3	0.6	0.36
Average shear stress on the shear surface (Pa) as obtained by simulations	293.95	44.96	79.55	124.85
Maximal shear stress on the shear surface (Pa)	1843.97	469.95	778.44	1007.12
*Legionella* number of colony—control (CFU/mL) as measured experimentally	–	2973	2973	2973
*Legionella* number of colony—handled (CFU/mL) as measured experimentally	–	535	380	750
Reduction rate (%)	99.98	82.00	87.22	74.77

In *BCR* experiments, colonies were obtained after one treatment, while in *SCR* experiments, six treatments were required. It is likely that several conditions must be met for a nearly 100% reduction in the number of cases. Furthermore, it is important that the cavitation number (Fig. 6) is close to the supercavitation range (0.6–0.7), and the value of the shear stress must also be high. In addition, the flow conditions of *SCR* had more favourable parameters in terms of reduction because, at a lower flow rate, the amount of sample to be treated was also smaller. Figure 6 also shows that the maximum reduction rate in the *BCR* design was at the intersection of the cavitation number and the shear stress, which could be the operating point of the system. It is possible that increasing the value of the shear stress in *BCR* already reached a threshold; thus, changing the flow and system parameters (increasing the speed using an asynchronous electric motor) would no longer result in an increase, except by changing the geometry. From the values, it was concluded that the “positive” geometric design was more efficient in terms of shear stress.

In “Boundary conditions and simplified simulation models” section, the target values on boundary conditions were described, where the torque measurement on the rotational cavitation components was defined. The software calculates the torque in the *x*, *y*, and *z* directions from the flow on the rotational cavitation-generating components, excluding the losses (e.g., friction).

Another reliable and easy way to check the simulation results is to measure the current consumption. The current consumption varies as a function of the torque on the electric motor shaft. The higher the load on the shaft of the electric motor is, higher is the current consumption. From the current consumption, the power and torque of the electric motor can be calculated in the case of a three-phase system, as given by Eqs. (8), (9).

8 $$P= \sqrt{3}UIcos\varphi$$

9 $$M= \frac{P}{\omega }$$

where *P* is the power of electric motor [W], *U* is the electrical voltage [V], *I* is the current [A] consumption, *cosφ* is the power factor, *M* is the torque [Nm] of the motor, and *ω* [rot/min] is the speed of the motor.

**Figure 8:**
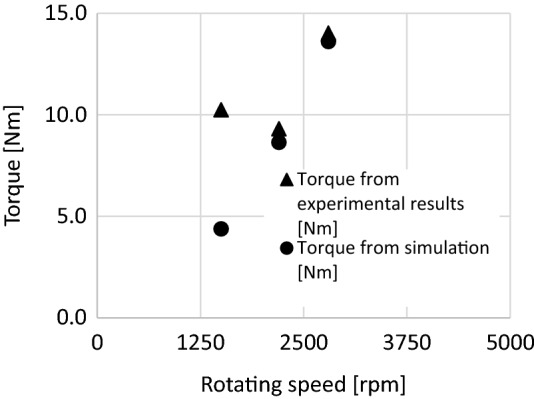
Torque comparison in BCR geometry.

From Fig. 8, it can be stated in general that the simulation torque results were lower in all cases than the real results. This can be an important consideration when selecting an electric motor, and a safety factor can be used to size the electric motor.

The relative errors can be seen in Table 4. The simulation results correlate well with the measured values.

**Table 4:** Relative error of simulation.

	SCR	BCR		
Rotating speed (rpm)	9025	1500	2200	2800
Torque from experiment results (Nm)		10.2	9.3	14.0
Torque from simulation (Nm)		4.4	8.6	13.6
Relative error (%)		57.2	7.2	2.9

With additional mesh compression, the results would probably give an even more accurate picture of the torque value, but this requires more computational time; however, a safety factor can also serve as a sizing principle. The largest deviation (with a relative error of 57.2%) was observed at 1500 rpm, the reason for which is currently unknown, and further investigations are needed to explore the problem.

## Conclusions

In this study, two reactors with different geometric designs generating a cavitation field were investigated using CFD simulation. Experimental validation was performed by measuring the reduction rate in *Legionella* colonies, and a satisfactory correlation was established with the numerical methods. In general, to reduce the *Legionella* colony count by up to 100%, several conditions must be met, including appropriate flow conditions, correct setting of the cavitation number, and shear stress, which largely depend on the geometric design of the reactor. The advantage of *SCR* was the high shear stress value, while a maximum working point was determined for *BCR* equipment. In the development of *BCR*, the goal was to increase the value of the shear stress by examining different geometries. The other advantage of *BCR* was that of the commercially available equipment, the motor had low power that was suitable for treating higher volume flows than SCR with a significant *Legionella* reduction rate. Furthermore, due to the different flow rates, more simulation would result in efficient values when handling larger, industrial-scale systems.

## Data Availability

All data generated or analysed during this study are included in this published article.

## List of symbols

*u*: Fluid velocity, m s^−1^
*ρ*: Fluid density, kg m^−3^
*S*_*i*_: Mass-distributed external force per unit mass, N kg^−1^
*g*_*i*_: Gravitational acceleration component, m s^−2^
*h*: Thermal enthalpy, J
*Q*_*H*_: Heat source per unit volume, W m^−3^
*τ*_*ik*_: Viscous shear stress tensor, N m^−2^
*q*_*i*_: Diffusive heat flux, W m^−2^
*ω*: Angular velocity of the rotating coordinate system, s^−1^
*r*: Distance from a point to rotation axis, m
*k*: Kinetic energy of turbulence, J kg^−1^
*h*_*m*_: Thermal enthalpy of *m*, J
*y*_*m*_: Concentration of component in the mixture, kg (solute) (solvent)^−1^
*µ*: Viscosity of fluid, kg m^−1^ s^−1^ (Pa s)
*µ*_*t*_: Turbulent viscosity, m^2^ s^−1^
